# Structural Analyses of Designed α-Helix and β-Sheet Peptide Nanofibers Using Solid-State Nuclear Magnetic Resonance and Cryo-Electron Microscopy and Introduction of Structure-Based Metal-Responsive Properties

**DOI:** 10.3390/ijms25021111

**Published:** 2024-01-16

**Authors:** Shota Nakagawa, Minami Kurokawa, Ohki Kambara, Toshiaki Takei, Kengo Daidoji, Akira Naito, Mao Takita, Akihiro Kawamoto, Mika Hirose, Atsuo Tamura

**Affiliations:** 1 Graduate School of Science, Department of Chemistry, Kobe University, Kobe 657-8501, Japan; 2083284s@stu.kobe-u.ac.jp (S.N.); 191s205s@stu.kobe-u.ac.jp (M.K.);; 2 Graduate School of Engineering, Yokohama National University, Yokohama 240-8501, Japannaito@ynu.ac.jp (A.N.); 3 Institute for Protein Research, Osaka University, Suita 565-0871, Japan; kawamoto@protein.osaka-u.ac.jp (A.K.); mhirose@protein.osaka-u.ac.jp (M.H.)

**Keywords:** nanofiber, peptide design, self-assembly, α-helix

## Abstract

The 21-residue peptide α3, which is artificially designed and consists of three repeats of 7 residues, is known to rapidly assemble into the α-helix nanofiber. However, its molecular structure within the fiber has not yet been fully elucidated. Thus, we conducted a thorough investigation of the fiber’s molecular structure using solid-state NMR and other techniques. The molecules were found to be primarily composed of the α-helix structure, with some regions near the C- and N-terminal adopting a 3_10_-helix structure. Furthermore, it was discovered that β-sheet hydrogen bonds were formed between the molecules at both ends. These intermolecular interactions caused the molecules to assemble parallelly in the same direction, forming helical fibers. In contrast, we designed two molecules, CaRP2 and βKE, that can form β-sheet intermolecular hydrogen bonds using the entire molecule instead of just the ends. Cryo-EM and other measurements confirmed that the nanofibers formed in a cross β structure, albeit at a slow rate, with the formation times ranging from 1 to 42 days. To create peptide nanofibers that instantaneously respond to changes in the external environment, we designed several molecules (HDM1-3) based on α3 by introducing metal-binding sites. One of these molecules was found to be highly responsive to the addition of metal ions, inducing α-helix formation and simultaneously assembling into nanofibers. The nanofibers lost their structure upon removal of the metal ion. The change occurred promptly and was reversible, demonstrating that the intended level of responsiveness was attained.

## 1. Introduction

Fibrous proteins such as keratin, collagen, and spider silk are abundant in nature. The molecules of these proteins form specific higher-order structures to perform their functions [1,2,3]. By referring to such natural fibrous proteins and controlling the self-assembling ability of protein molecules, it is possible to artificially create fibers with desirable structures and functions. To explore this possibility, efforts are currently underway to artificially design peptides that form nanofibers and allow for control of their structures and functions at the nano level.

Pioneering studies on creating desired structures with peptide nanofibers include those by Woolfson [4] and Conticello et al. [5]. Similarly, as an example of the attempts to add functionality, artificially designed fibers that can function as tissue regeneration agents or drug delivery system carriers have been reported [6,7]. These results demonstrate the advancement of the field in creating novel structures and functions of peptide nanofibers. Here, we present advanced structural analyses of α-helical and β-sheet nanofibers using the designed peptides. We then aim to achieve metal ion responsiveness through molecular design based on the structural analysis at the atomic level.

The amino acid sequences of the six designed peptides used in this study are shown in Table 1.

**Figure 1:**
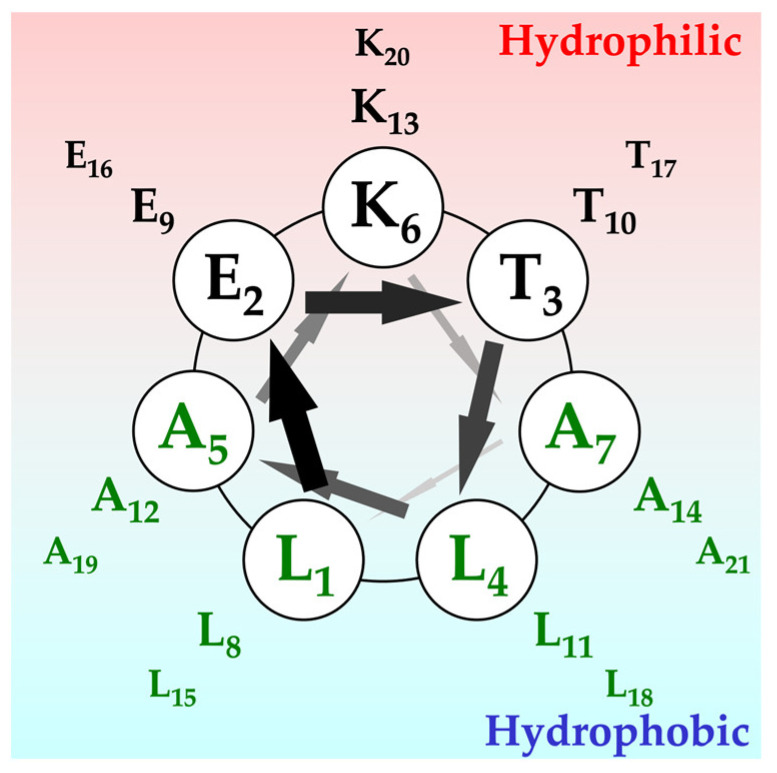
Helical wheel representation of the α3 peptide. The primary sequence of α3 peptide is three times repeated Leu-Glu-Thr-Leu-Ala-Lys-Ala. Upper side of the wheel consists of hydrophilic residues, like Lys, Glu, and another hydrophobic surface by Leu residue.

For the α-helix-type fiber, we focused on the α3 peptide [8,9,10,11] designed by Kojima et al. The α3 peptide is an amphiphilic peptide consisting of 21 residues in which the L-E-T-L-A-K-A sequence is repeated three times (Figure 1).

The peptide is known to form fibers 5–10 nm wide and several μm long at neutral pH. Although the peptide is known to form a protofibril as a tetramer before assembling into the fiber according to the sedimentation equilibrium experiments, its atomic level structure has not yet been fully elucidated.

Therefore, in this study, we attempted to elucidate the structure of the fiber formed by the α3 peptide by using polarized infrared microscopy and ^13^C solid-state NMR spectroscopy, which can be measured in the solid state without being hampered by the sample morphology or molecular weight increase caused by aggregation. As a result, it was found that the α3 molecules assembled into the fiber by forming β-sheet-like hydrogen bonds between the terminal sites of the tetrameric units consisting mainly of the α-helix, which were elongated in the fiber axis direction. Amyloid fibers are a typical example of proteins that form fiber-like proteins via hydrogen bonds in the β-sheet structure [12,13]. We then additionally designed CaRP2, consisting of 13 residues, and βKE, consisting of 7 residues, both of which were expected to form intermolecular β-sheet hydrogen bonds throughout the molecule and assemble into amyloid-like fibers. Cryo-EM observations were performed on the fibers formed by these peptides for structural analyses. Next, we attempted to confer the metal-binding ability to the α3 peptide, which formed fibers more rapidly than β-sheets. Based on the structural information of α3, we introduced His residues [14], amino acids with a high binding ability to metal ions, at appropriate positions, anticipating the ability to selectively respond to the type of metal ion added and to switch the fiber-forming ability on and off. The three candidate peptides (HDM1, HDM2, and HDM3) are listed in Table 1. The binding ability of these peptides to divalent metal ions (Co^2+^ and Ni^2+^) was evaluated. As a result, we succeeded in creating peptide nanofibers with a switching fiber-forming ability depending on the addition of Ni^2+^ for HDM1. HDM1 nanofibers are expected to have high industrial application value in fields such as engineering and medicine, serving as metal-sensing materials and biomarkers.

## 2. Results and Discussions

### 2.1. Secondary Structures of α3 and Their Orientation

**Figure 2:**
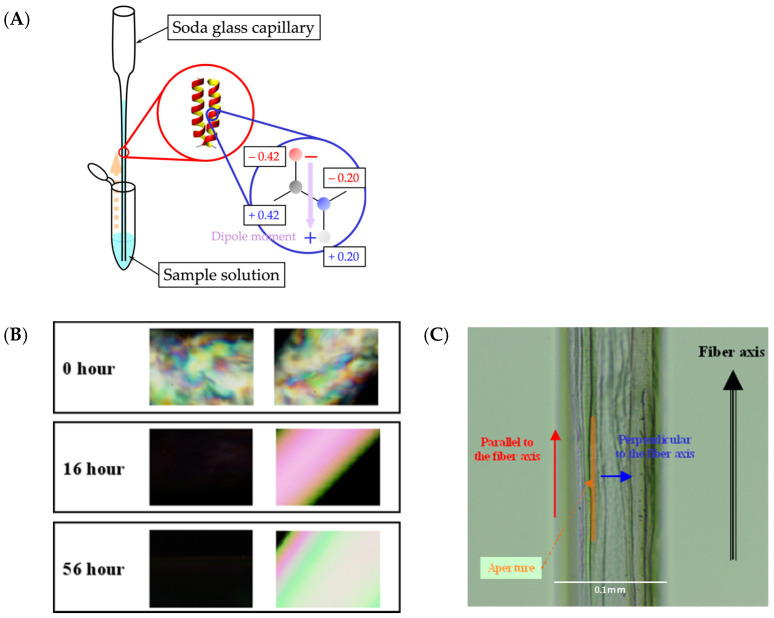
Sample preparation and morphological observation of the α3 peptide for polarized IR measurements. (**A**) After transferring the sample solution to the capillary, it was placed in a magnetic field of 17.4 T, which corresponds to a proton resonance frequency of 750 MHz. The α-helices are aligned in the direction of the magnetic field. (**B**) Polarized-light microscope images after 0, 16, and 56 h of standing in a magnetic field. (**C**) Optical microscope image of the lyophilized sample taken at the 56 h mark. The blue and red arrows represent the directions parallel and perpendicular, respectively, to the fiber axis.

The α-helix exhibits magnetic susceptibility due to the planar structure of its pi electrons. The orientation of the pi electrons, which is the direction of the helix, is perpendicular to the magnetic field direction as the induced magnetic dipole densities of pi electrons are most stabilized when the induced magnetization is minimized. Then, the initial step in preparing the sample for the polarized IR-microscope measurements involves incubating it in a high magnetic field (Figure 2A,B) confined in a capillary. To eliminate any interference from H_2_O that may disturb the secondary structure analysis in the amide I’ region of the IR spectra, the aligned sample was lyophilized (Figure 2C).

**Figure 3:**
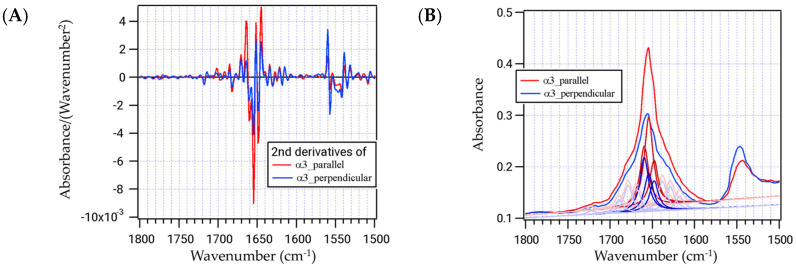
Polarized infrared spectra of the α3 peptide, without isotopic labeling. (**A**) The second-order differential spectra of α3 parallel (red) and perpendicular (blue) to the axis are shown. (**B**) Deconvolution of the IR spectra for the parallel (red) and perpendicular (blue) directions. Major components are in dark lines and minor components are in thin lines.

Polarized infrared spectroscopy was performed on the samples of the unlabeled α3 peptides thus prepared. The second derivative IR spectra of the α3 peptide exhibited three large negative local minimum bands around the amide I’ region (Figure 3A). The wavelengths of these three peaks are 1648, 1654, and 1660 cm^−^^1^, and the secondary structures corresponding to these peaks are the random coil, α-helix, and 3_10_-helix or type III turn, respectively [15,16]. The 3_10_-helix can be formed by a series of type III turns, making these two structures almost identical and indistinguishable. It is to be noted that the type III turn is currently classified as one of the type I turns [17]. Deconvolution of IR spectra is expected to reveal each constituent secondary structure of the α3 peptide, and the polarized IR-microscope method can clarify the orientation of each constituent from the direction of the magnetic field. The IR spectra were deconvoluted and reconstituted using the Lorentz equation with a half-width of 6 cm^−^^1^ (Figure 3B).

When the peptide is irradiated with polarized light, the infrared absorption becomes maximum in the C=O alignment direction (parallel to the magnetic field) and minimum in the perpendicular direction. After deconvolution of the spectra of each direction, it was evident that the areas at 1648, 1654, and 1660 cm^−^^1^ were 1.66, 3.28, and 2.21 for the parallel polarized light and 1.12, 1.41, and 1.99 for the perpendicular light, respectively (Table 2). Then, the dichroic ratios, (area parallel)/(area perpendicular), of these three bands can be calculated to be 1.48 (1648 cm^−^^1^), 2.33 (1654 cm^−^^1^), and 1.11 (1660 cm^−^^1^) (Table 2). The value at 1654 cm^−^^1^ for the α helix component is the largest because the C=O groups are dominant and aligned along the helix axis parallel to the fiber direction. The angles of the C=O bonds can be calculated from the following equation [18]:

$$R_{\left(\alpha ,f\right)}=\frac{fcos^{2}\alpha +\frac{1}{3}\left(1-f\right)}{\frac{1}{2}f\sin^{2}\alpha +\frac{1}{3}(1-f)}$$

where *R*(*α*,*f*) is the dichroic ratio, *α* is the requiring angle, and *f* is the order parameter calculated as ([dichroic ratio] − 1)/([dichroic ratio] + 2). The angles thus calculated for the three constituents are 37.3°, 0°, and 50.2°, respectively (Table 2).

### 2.2. Secondary Structures and Distances between ^13^C and ^15^N of α3 Peptide Revealed by ^13^C Solid-State NMR

To further elucidate the microscopic structure at the atomic level, we prepared two types of isotope-labeled peptides, one synthesized with a single ^13^C=O-labeled amino acid and the other synthesized with both ^13^C=O and ^15^N doubly labeled (Table 3). The first column of the table contains the residues in the terminal regions, while the second and third columns contain the residues in the middle part.

**Figure 4:**
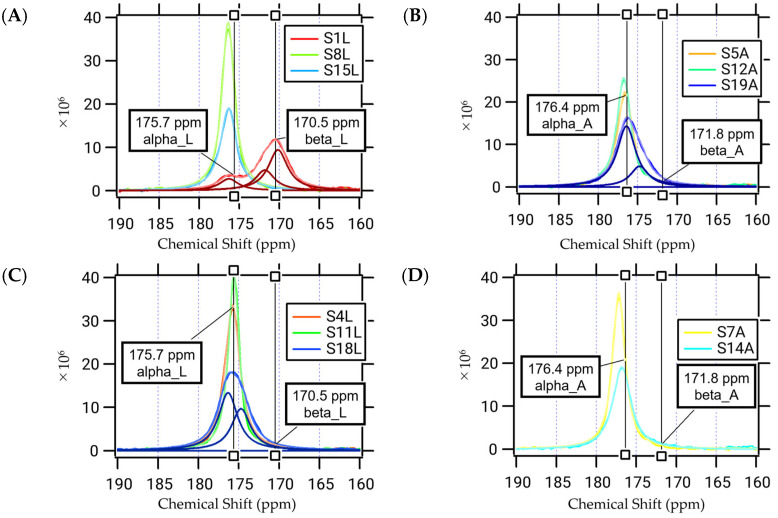
Solid-state CP-MAS ^13^C-NMR spectra of the ^13^C-labeled α3 peptides. Each carbon atom in C=O, specifically those at (**A**) Leu 1, 8, or 15; (**B**) Leu 4, 11, or 18; (**C**) Ala 5, 12, or 19; and (**D**) Ala 7 or 14, was labelled with ^13^C. Chemical shifts of Leu for α-helix (175.7 ppm) and β-sheet (170.5 ppm) and Ala for α-helix (176.4 ppm) and β-sheet (171.8 ppm) are presented in the figure. Deconvolutions were carried out for S1L (**A**), S19A (**B**), and S18L (**C**).

The ^13^C CP-MAS spectra of the individual α3 peptides labeled with a single ^13^C atom are anticipated to elucidate the secondary structure at the respective labeled position by examining the chemical shift values [19,20]. The resulting chemical shifts corresponding to the middle region of the peptide shown in Figure 4B–D can be attributed to the α helix for Leu4, Leu8, Leu11, and Leu15 at 175.7 ppm and Ala5, Ala7, Ala12, and Ala14 at 176.4 ppm. However, the spectra of the remaining three ^13^C-labeled positions at each terminal region, Leu1, Leu18, and Ala19, do not conform to a single Lorenz curve, necessitating two or three components as illustrated in Figure 4A. In fact, Leu18 can be analyzed using two components at 176.3 and 174.7 ppm, both of which can be assigned to the α-helix. However, they suggest a multiconformational nature or loss of stability of the helical structure. This trend was more pronounced for Ala19. The two peaks correspond to 176.4 and 174.7 ppm, with the latter located in between the α helix (176.4 ppm) and β-sheet (171.8 ppm). At the N-terminal, Leu1 can be fitted by three Lorenz curves at 176.3, 171.8, and 170.2 ppm, with an integrated ratio of 3:7:10. Notably, the percentage of the α-helix (176.3 ppm) is the lowest, while that of the β-sheet (170.2 ppm) is the highest. This suggests that the “fibro-overlap” segment of the α3 peptide may form a β-sheet-type hydrogen bond between the N-terminus of one molecule and the C-terminus of the other.

### 2.3. Intermolecular Distance between α3 Molecules

Four ^13^C- and ^15^N-double-labeled α3 peptides were prepared for the REDOR experiments: Leu1[^13^C]-Ala5[^15^N] and Leu15[^13^C]-Ala19[^15^N] for the determination of the intramolecular distances and Leu1[^13^C]-Ala21[^15^N] and Leu1[^15^N]-Ala21[^13^C] for that of the intermolecular distances (Table 3). The ^13^C-^15^N internuclear distances were evaluated by analyzing the plot of S/S_0_ against NcTr (Equation (1)) [21]. The distance between Leu1[^13^C]-Ala5[^15^N] and Leu15[^13^C]-Ala19[^15^N] was found to be 4.6 Å and 4.5 Å, respectively. These values exceed the normal distance of 4.0 Å between C and N in the α helix, indicating a partial collapse of the α helix structure at both ends of the α3 peptide. This observation is consistent with previous results obtained from the CP-MAS NMR measurements. Likewise, the REDOR experiment revealed that the distances between Leu1[^13^C]-Ala21[^15^N] and Leu1[^15^N]-Ala21[^13^C] were 4.7 and 5.1 Å, respectively. These measurements should reflect intermolecular distances because they are significantly smaller than the anticipated intramolecular distance between Leu1 and Ala21. Furthermore, they demonstrate that the molecules are oriented parallel to one another when aligned in the same direction, rather than antiparallel. 

### 2.4. Structure of α3 Peptide Nanofiber

**Figure 5:**
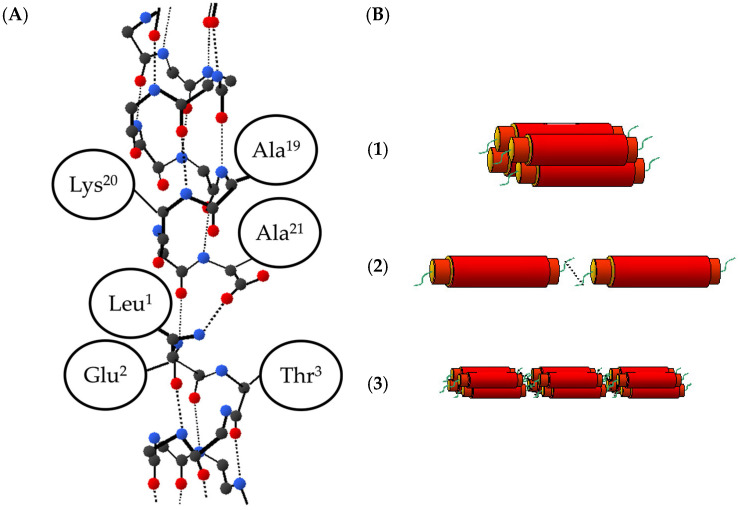
(**A**) Possible structural model for the α3 peptide. Carbon, oxygen, and nitrogen atoms are represented by black, red, and blue circles, respectively, while covalent bonds are depicted in solid lines and hydrogen bonds in dashed lines. Side chains of amino acid residues are simplified as ellipses. It is likely that the hydrogen bond of each peptide terminus has collapsed to some extent, leading to a 3_10_-helix in that region. The hydrogen bond between 1L[^13^C] and 21A[^15^N] is formed as a β-sheet type. (**B**) Schematic drawing of the fibrotic process. (1) Four monomeric α3 peptides gather in parallel together by the hydrophobic effect in the aqueous solution. (2) When the C- and N-terminal of α3 protofibril come closer, each protofibril is connected to each other by the β-sheet-type hydrogen bond. (3) Consequently, the solid fiber of α3 peptide is formed.

Based on the above IR and NMR results, it is evident that the peptide α3 exhibits a complete α-helix structure in the midsection (Leu4 to Leu15), while the helix structure is destabilized near Leu18 in the C-terminal region, resulting in a polymorphism with the 3_10_-helix. Moreover, β-sheet-type hydrogen bonds are formed between the molecules near both ends (Leu1 and Ala19). The molecules align parallel to each other in the same direction, connected by β-sheet hydrogen bonds at both ends (Leu1 and Ala21). Figure 5A shows a schematic drawing of this structural model consistent with the information provided.

The 3_10_-helix structure located close to the peptide’s end is less stable and more elongated when compared to the α helix structure, which is occasionally found in nature. In the case of α3 peptides, the flexibility of the helical structure thus induced may have facilitated intermolecular interactions, leading to the formation of β-sheet hydrogen bonds and ultimately fibrillation. Therefore, fibrillation is considered to proceed as depicted in Figure 5B. Initially, in an aqueous solution, a monomeric α3 peptide folds into a “protofibril”, which comprises four peptides [8]. Next, as the C- and N-termini of the α3 protofibrils approach each other, they are interconnected by β-sheet-type hydrogen bonds. Finally, a robust α3 peptide fiber is formed.

Statistical studies on the protein structure have indicated that in motifs transitioning from α-helix to β-strand, about 33% take the 3_10_-helix structure at the end of the α-helix [22]. This fact is also consistent with the so-called helix nucleation scheme, which proposes that the formation of an isolated β-turn is a helix nucleation step in the process of protein folding [23]. This reinforces the β-type hydrogen-bond network of the fiber structure of the α3 peptide and the suggested pathway for fiber formation presented in Figure 5. Additionally, it is known that the dihedral angles Φ and Ψ of the type III turn are identical to those of the 3_10_-helix. Therefore, it is structurally reasonable to assume that the terminal portion of the α-helix is elongated to form a 3_10_-helix, and a β-sheet-like hydrogen bond is formed in the type III turn manner between molecules. Note that although type III is currently incorporated into type I as a classification for β-turns and is not treated separately [17], it is discussed here due to its distinctive dihedral angles.

Kurokawa et al. compared the thermostability of fibers formed by α3CB peptides, in which the C-terminus of the α3 peptides is amidated, with that of the α3 counterparts [24]. The results indicated that the amidation increased the thermal transition temperature by more than 10 °C. The hydrophobic moment was then investigated as an indicator to identify the underlying reason for the observed improvement, which revealed the enhanced hydrophobicity in the C-terminal direction of the helix molecule. Based on the structural information obtained for the α3 fibers in this study, the interpretation of the improvement in thermal stability is that the amide modification of the negatively charged carboxylic groups reduces polarity, resulting in a relative increase in hydrophobicity at the C-terminus of the tetrameric units. Consequently, this enhances the strength of the β-sheet-type hydrogen bonds between the units, resulting in the enhanced thermal stability of the α3CB peptide fibers.

### 2.5. Structural Analyses of β-Sheet-Type Peptide Nanofibers

The structural analyses of the α3 fiber revealed that the α3 molecules form β-sheet-like hydrogen bonds, enabling intermolecular association and elongation along the fiber axis (Figure 5). In the aggregated states of the amyloids, although β-sheet hydrogen bonds predominate the intermolecular interactions, some retain an α-helical conformation in their assemblies [25,26]. Thus, the formation of the intermolecular β-sheet bonding can be regarded as crucial for fibrilization. Therefore, we designed two peptides to form amyloid-like fibers by adopting β-strands and forming β-sheet-type intermolecular hydrogen bonds throughout the molecule. Subsequently, we analyzed the structures of these peptides, whose sequences are listed in Table 2. CaRP2 was designed to improve the β-sheet and β-turn properties of the EF3 region (Y109-E121) of a calcium ion-binding protein called Recovelin [PDB ID:4YI8] [27]. In contrast, βKE is based on the β1 chain of the POIA1 protein [PDB ID: 1ITP] from a basidiomycete [28], and is based on the shortest fiber-forming sequence KFIVIFK, where the C-terminal K is substituted with E to enhance its forming ability. Both peptides assumed a β-sheet conformation, resulting in the formation of a homogenous fiber that is suitable for further structural analysis. We then proceeded to examine the fiber structures created by both peptides.

**Figure 6:**
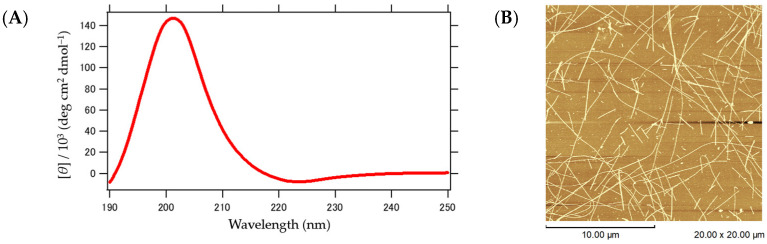

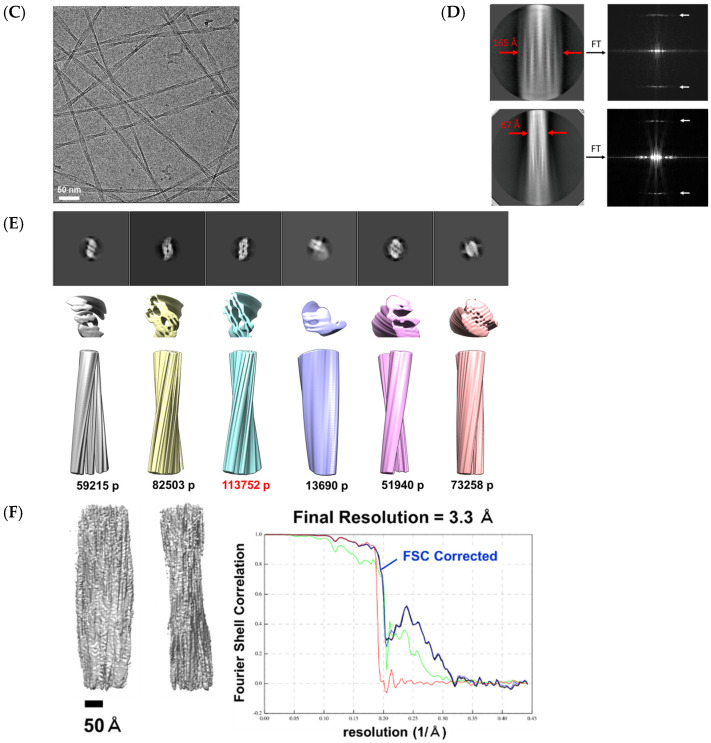
Structural characteristics of the CaRP2 fiber. (**A**) CD spectrum measured at 25 °C. (**B**) AFM image observed at 25 °C with a scale bar of 10.00 μm. (**C**) Cryo-EM observation with a scale bar of 50 nm. (**D**) A section of 2D classification result (left)/Fourier transform of the 2D classification images (right). (**E**) Three-dimensional classification results. (**F**) Three-dimensional reconstructed structure model and Fourier Shell Correlation graph. The blue curve is the FSC-Corrected function. (Black: rlnFourierShellCorrelation, green: rlnFourierShellCorrelationUnmaskedMaps, blue: rlnFourierShellCorrelationMaskedMaps, and red: rlnCorrectedFourierShellCorrelationPhaseRandomizedMaskedMaps).

**Figure 7:**
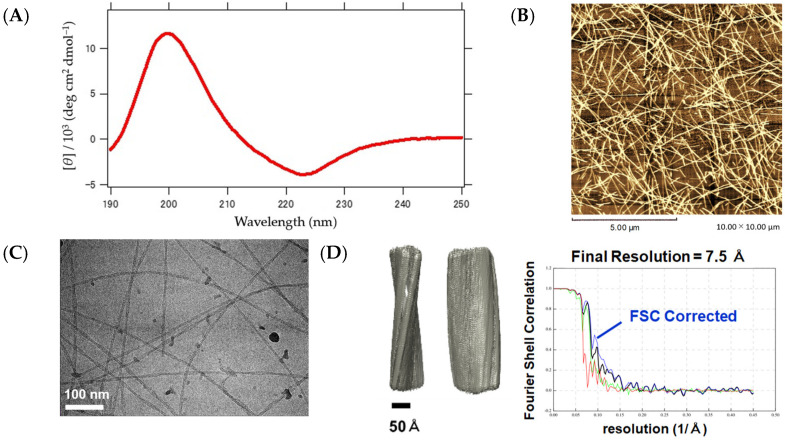
Structural characteristics of the βKE fiber. (**A**) CD spectrum measured at 25 °C. (**B**) AFM image observed at 25 °C with a scale bar of 5.00 μm. (**C**) Cryo-EM observation with a scale bar of 100 nm. (**D**) Three-dimensional reconstruction model and Fourier Shell Correlation graph. The blue curve is the FSC-Corrected function. (Black: rlnFourierShellCorrelation, green: rlnFourierShellCorrelationUnmaskedMaps, blue: rlnFourierShellCorrelationMaskedMaps, and red: rlnCorrectedFourierShellCorrelationPhaseRandomizedMaskedMaps).

Figure 6A–F presents the results of the structural analysis of CaRP2. The CD measurements showed a positive peak at around 202 nm, indicating the formation of β-turn and β-pleated structures (Figure 6A). This positive peak was exceptionally large, suggesting the presence of eminent fiber twists [29]. Furthermore, the AFM measurements confirmed that the CaRP2 peptide formed long, rigid, and homogeneous fibers (Figure 6B). The cryo-EM observation also revealed twisted flat fibers (Figure 6C), which is consistent with the AFM observation. Additionally, the 2D classification of the image in Figure 7C (Figure 6D, left) followed by Fourier transformation revealed equally spaced diffraction lines at 4.7 Å (Figure 6D, right). This value is identical to the distance between the β-strands in the β-sheet structure. The 3D classification was conducted using the 2D classification images, resulting in the successful generation of a 3D model with a resolution of 3.3 Å (Figure 6E,F). These results suggest that the peptide adopts β-strands with a β-turn structure and interacts with other molecules through β-sheet-type hydrogen bonding, ultimately leading to the formation of an amyloid-like fiber with a cross β structure.

**Figure 8:**
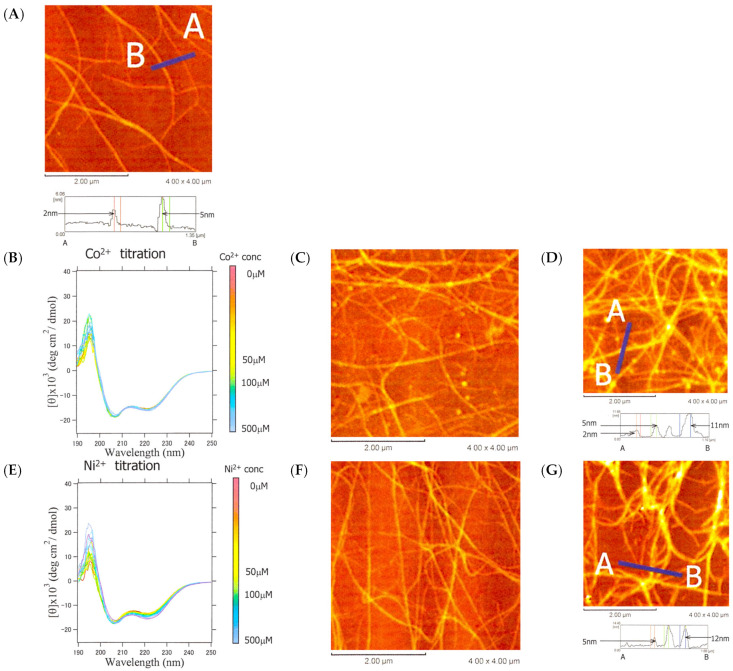
CD spectra and AFM images of α3 during the titration with metal ions. (**A**) AFM image of α3 (100 μM in 5.56 mM HEPES buffer pH 7.6). (**B**) Change in CD spectra of α3 upon addition of Co^2+^. (**C**) AFM image when 50 μM of Co^2+^ is added. (**D**) AFM image when 100 μM of Co^2+^ is added. (**E**) Change in CD spectrum upon addition of Ni^2+^. (**F**) AFM image when 50 μM of Ni^2+^ is added. (**G**) AFM image when 100 μM of Ni^2+^ is added.

Next, the results of the structural analysis of the βKE fiber are presented in Figure 8A–D. The CD measurement showed a spectrum (Figure 7A) similar to that of the seven-residue synthetic peptides Boc(Ala)7-OMe or Boc(Val)7-OMe, which form the β-sheets [30]. The morphology was observed using AFM (Figure 7B) and cryo-EM (Figure 7C), both of which displayed nearly straight, loosely curved, and uniformly elongated fibers. After the 2D classification and Fourier transform of the cryo-EM images, diffraction stripes spaced equally at 4.8 Å were observed. This value is also in accordance with the distance between the β-strands, confirming the involvement of the β-sheet architecture in the intermolecular structure. Additionally, a 3D model was successfully created at a resolution of 7.5 Å based on the 2D classification images (Figure 7D). These results demonstrate that βKE, like CaRP2, possesses a cross β structure, in which the layers are elongated along the fiber axis via hydrogen bonding. It should be noted that while variants of βKE, such as βEK and βDK, also showed the formation of β-sheet fibers according to the AFM and CD observations, the cryo-EM analysis was not successful, presumably due to their inhomogeneity.

### 2.6. Introducing Conformational Switching Capability upon Metal Ion Binding

The formation of the β-sheet fibers in the preceding chapter progressed remarkably slowly, with CaRP2 requiring approximately 42 days and βKE 1 day. In contrast, the formation of the α3 helix fibers occurred instantaneously upon sample preparation. Thus, when peptide nanofibers are used as functional materials, the use of α helix fibers is expected to confer a rapid structural switching ability. We chose to redesign α3 in order to quickly form a structure via metal binding and lose the structure via metal dissociation. As a prerequisite, we investigated the metal-binding properties of α3 itself.

The AFM observation revealed that α3 consisted of a mixture of fibers with a height of approximately 2 nm (Figure 8A), corresponding to a tetrameric helix bundle (Figure 5B-3), and fibers of approximately 5 nm, which may have been formed by twisting the tetrameric bundle together. The addition of Co^2+^ or Ni^2+^ did not significantly affect the helicity, as shown by the CD, but the AFM image indicated an increase in height to 11–12 nm, suggesting further assembly and twisting of the fibers (Figure 8B–G). In other words, the metal ions only had the effect of bundling more fibers together and did not contribute to the helix structure itself.

We then attempted to design mutants in which structural switching was induced by the addition of metal ions. Our objective is to cause conformational switching upon the addition of metal ions, resulting in a change in the secondary structure, followed by the further association of molecules to form higher-order structures. To achieve this, we designed a series of mutants that satisfies two conditions: (1) The peptide is unstructured, i.e., random coiled, in the absence of metal ions. (2) The addition of a metal ion induces the peptide to form an α-helix, which further assembles into a fiber. Histidine was chosen as the amino acid residue that binds to the metal ion. Two positions were selected among the hydrophilic residues exposed to the solvent to introduce the His residues. The two His residues should be located in close proximity when forming the α-helix structure to allow the metal ion to be trapped between them. To fulfill condition (1), charged Lys or Glu was replaced with His so that the helix structure could not be maintained by electrostatic repulsion. Three α3 mutants were thus designed while considering the aforementioned conditions. They were named HDM1, -2, and -3, and their amino acid sequences in the helical wheel representation are shown in Figure S1A–C.

### 2.7. Structure and Metal Ion Response of HDM1, -2, and -3

**Figure 9:**
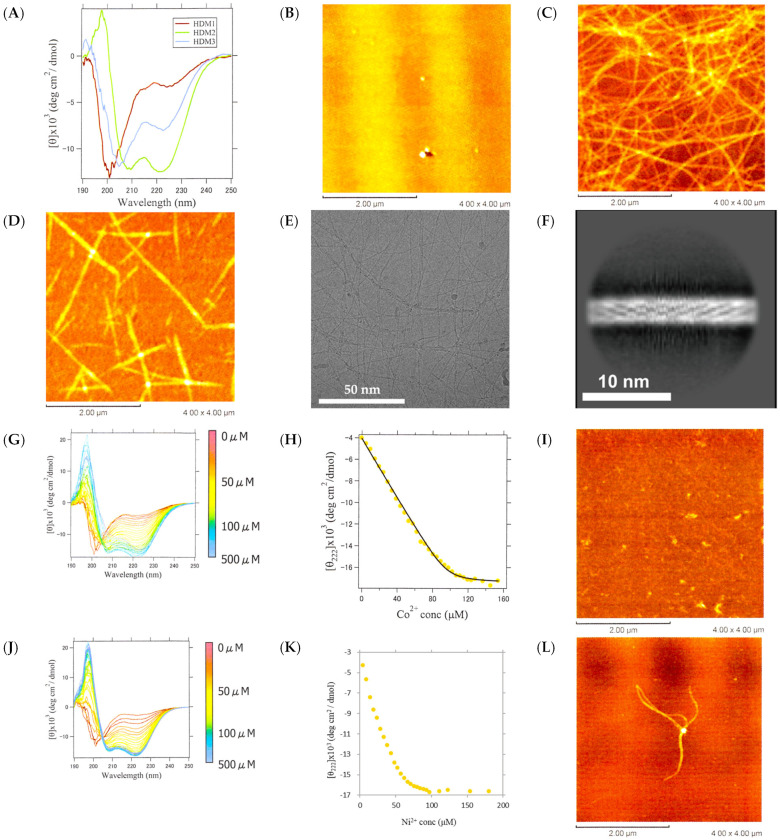
Structural observations of HDM1-3. (**A**) CD spectra of HDM1-3 at 25 °C. AFM images of (**B**) HDM1, (**C**) HDM2, and (**D**) HDM3 (scale bar 2.00 μm). (**E**) Cryo-EM image of HDM3 fiber (scale bar 50 nm), and (**F**) 2D averaged image of HDM3 fiber obtained via cryo-EM (scale bar 10 nm). (**G**) CD spectra of HDM1 with varying concentrations of Co^2+^. (**H**) The change in [θ]_222_ and fitting by the theoretical curve, and (**I**) AFM image after addition of 100 μM of Co^2+^ to HDM1. (**J**) CD spectra of HDM1 with varying concentrations of Ni^2+^. (**K**) The change in [θ]_222_, and (**L**) AFM image of HDM1 after addition of 100 μM of Ni^2+^.

The CD measurements at pH 7.6 revealed that HDM1 had a random coil structure, while HDM3 exhibited spectra characteristic of the α-helix, and HDM2 was somewhere in between (Figure 9A). According to the AFM measurements, fibers were observed for HDM2 and HDM3 (Figure 9C,D). The fibers of HDM3 showed excellent homogeneity and linearity, which allowed us to observe them via cryo-EM (Figure 9E,F). Although the Fourier transform of Figure 9F did not produce a clear diffraction pattern, the 2D average image (Figure 9F) differed significantly from the pattern of the β-sheet fibers of CaRP2 shown in Figure 6D. Instead, it displayed helical stripes similar to the 2D average image of α-helical fibers designed by Egelman et al. [31] These results suggest that HDM3 formed α-helical fibers that elongated in the direction of the helix axis.

HDM1 was the only peptide in the random coil conformation due to its different net charge (−2 at pH 7.6) compared to the other peptides. This difference in charge may have destabilized the structure due to electrostatic repulsion. To confirm this, we conducted a pH titration on HDM1-3 and α3. The results are presented in Figure S2A–L, along with the calculated net charge as a function of pH and the AFM images at the pH at which the fibers formed. Based on these results, when the net charge ranged from −1 to +1 pH, all the peptides exhibited the lowest ellipticity at 222 nm and fiber formation was confirmed by using AFM, indicating that the peptides formed helical fibers at a net charge near zero, where electrostatic repulsion is low.

At pH 7.6, HDM1 had a net charge of −2 and was unstructured. Upon binding with divalent metal ions, the net charge is expected to become zero, reducing electrostatic repulsion and forming helix fibers. Thus, Co^2+^ or Ni^2+^ was added to HDM1 (Figure 9G–L). The CD values at 222 nm were plotted against the concentration, and curve fitting was performed using the 1:1 binding model shown in Equations (4)–(6) (Figure 9H). The binding constant *K*_B_ was obtained to be 1.0 (±0.1) × 10^6^. However, the AFM measurements did not reveal any fibers, only granular aggregates (Figure 9I). Upon the addition of Ni^2+^, the ellipticity at 222 nm also decreased with an increasing metal ion concentration (Figure 9J). However, the simple 1:1 binding model did not yield a proper fitting curve (Figure 9K). This is likely due to the coexistence of the binding reaction between HDM1 and Ni^2+^ as well as a reaction that results in a multimeric structure. The AFM measurements did reveal a fibrous structure (Figure 9L). Similar results were obtained for Cu^2+^ and Zn^2+^ (Figure S3). Cu^2+^ titration showed a sigmoidal response, indicating cooperative metal binding, while Zn^2+^ bound stoichiometrically but weakly (*K*_B_ = 7.3 (±1.4) × 10^5^) compared to Co^2+^. These results suggest that HDM1 underwent a secondary structure transition from a random coil to an α-helix upon binding to divalent metal ions, with octahedral coordination being advantageous. Furthermore, the formation of a helical fiber was confirmed upon binding to Ni^2+^.

### 2.8. Identification of Ni-Binding Sites in HDM1

**Figure 10:**
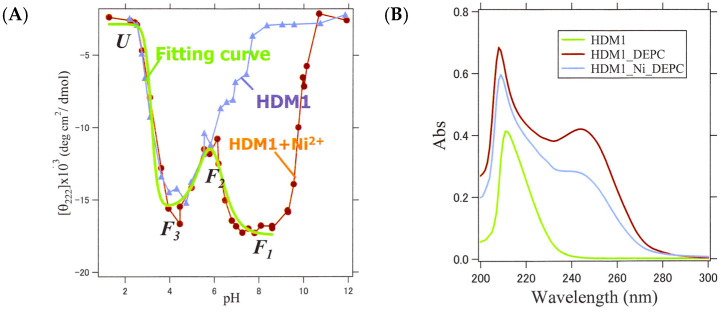

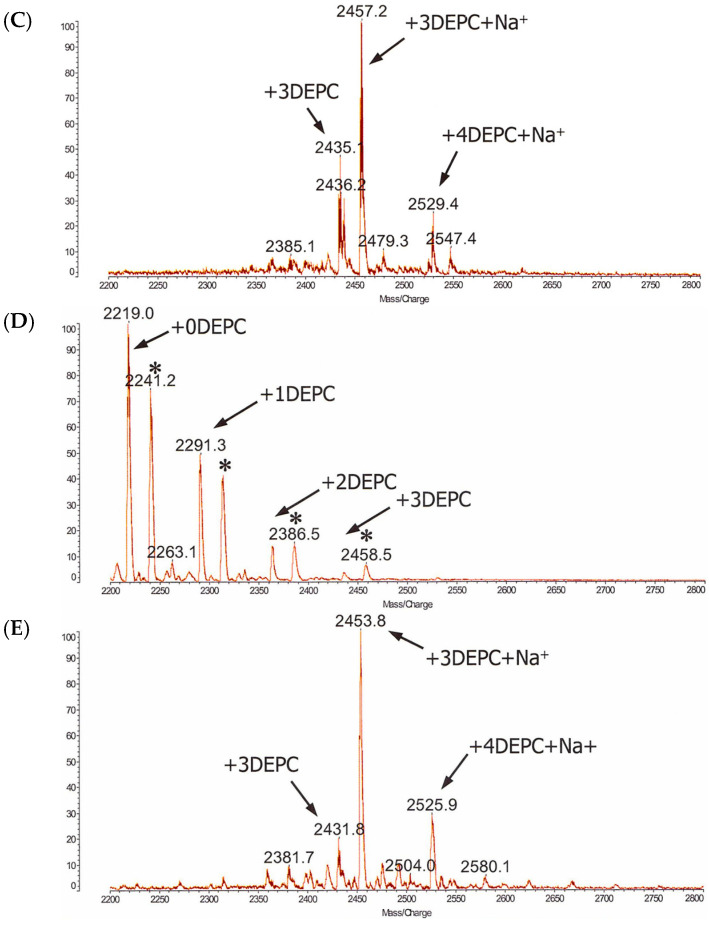
CD and MALDI-TOF-MS experiments to identify the Ni^2+^-binding site of HDM1. (**A**) CD values of HDM1 (blue) and HDM1+Ni^2+^ (red) measured at various pHs. The theoretical curve fit for the three-step transition with three pKa values is also displayed. (green). (**B**) UV spectra of HDM1, HDM1 after DEPC modification, and HDM1+Ni^2+^ after the modification. (**C**) MS spectrum of HDM1, which does not form fiber without metal ions, after DEPC modification. (**D**) MS spectrum of HDM1+ Ni^2+^ (* indicates Na+). (**E**) MS spectrum of HDM2, which can form fiber without the addition of metal ions.

The binding sites of Ni^2+^ were confirmed by two experimental methods. Firstly, we examined the pH dependence of CD to see if structural changes occur upon binding near the pKa of the His residue. We performed pH titration on HDM1 and HDM1-Ni^2+^ (molar ratio 1:1 mixture) and observed a change in the [θ]_222_ value of CD (Figure 10A). The results showed that the [θ]_222_ values for both samples decreased from acidic to pH 6 as the pH increased, and then those increased slightly. However, between pH 6 and neutral, the [θ]_222_ value continued to increase for HDM1, while it decreased again for HDM1-Ni^2+^, returning to the metal-bound α-helix structure. This suggests that a proton was deprotonated from a side chain when the pH exceeded 6, causing the side chain group to become uncharged and capable of binding metal ions. Given that the only amino acid residue with a pKa near 6 is His, it is highly likely that Ni^2+^ bound to the His residues.

Secondly, an experiment was conducted to chemically modify the nitrogen atoms in the His residues using DEPC (Figure 10B–E). It is estimated that for HDM1 at pH 7.6, the binding order will be His side chains, the N-terminal, and the Lys side chain [32]. However, it has been reported that DEPC is less likely to chemically modify His when His is coordinately bound to metal ions [33]. This property was utilized to identify the binding site through the combined use of mass spectrometry. UV spectroscopy confirmed the introduction of DEPC into HDM1 (Figure 10B), and the mass spectra were measured in the absence (Figure 10C) and presence (Figure 10D) of Ni^2+^. To account for the potential inhibition of DEPC modification via fiber formation, a similar experiment was conducted for HDM2, which can form fibers in the absence of metal ions (Figure 10E). Table 4 summarizes the assignments derived from these experiments. The metal-free fibers of HDM2 did not show the +5DEPC peak, indicating that DEPC was not introduced at the N-terminus, possibly due to the formation of α3-like intermolecular hydrogen bonds upon fiber formation. Additionally, the binding to His and Lys even after fiber formation suggests that both His and Lys are located outside of the fiber and can be modified. In contrast, HDM1 showed that all two His, N-terminal, and Lys were modifiable, and the major component was +3DEPC, excluding the less reactive Lys. On the other hand, HDM1-Ni^2+^ showed the main components of 0DEPC and +1DEPC. This can be explained by assuming that the two His residues are blocked by the bound Ni^2+^, while the N-terminal is unmodified in the fibrous state and Lys is intrinsically difficult to modify, as previously described. Overall, it is strongly suggested that His is evidently bound to Ni^2+^.

### 2.9. Conformational Switching Capability of HDM1 Fiber by Metal Ion

**Figure 11:**
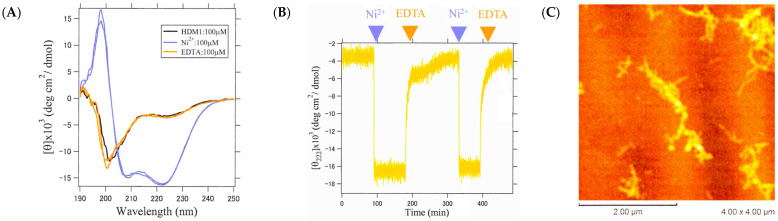
Change in the helix fiber structure upon repeated addition and removal of metal ions. (**A**) CD spectra of HDM1 alone (black), after the addition of Ni^2+^ (blue), after the addition of EDTA (orange), and once again after the addition of Ni^2+^ (blue). The near overlap of black, orange, and two blues indicates structural reversibility. (**B**) Time variation in [θ]_222_ when Ni^2+^ and EDTA were added alternately to HDM1. (**C**) An AFM image after adding EDTA and then Ni^2+^ again.

The reversibility of the conformational transition of the HDM1-Ni^2+^ fiber was investigated. For this purpose, the process of alternately adding EDTA to remove metal ions and then adding Ni^2+^ again was repeated several times, and the responses were observed through the CD and AFM measurements (Figure 11A–C). The results showed that the addition of EDTA caused a gradual loss of structure over several minutes, and the re-addition of Ni^2+^ caused an almost instantaneous formation of helical fibers (Figure 11B), whose process was reproducible multiple times. AFM measurements also confirmed the reversibility of fiber formation upon the re-adding of Ni^2+^ (Figure 11C). Therefore, it can be concluded that peptide fibers with a metal-selective conformational switching ability have been successfully designed.

## 3. Materials and Methods

### 3.1. Synthesis of Peptides

The peptides were synthesized by the solid-phase method. The synthesis was carried out on an automated peptide synthesizer Pioneer (PerSeptive Biosystems, Framingham, MA, USA), using continuous flow techniques, the Fmoc strategy, and polyethyleneglycol/polystyrene support. The functional groups of amino acids (Peptide Institute, Inc., Osaka, Japan) were protected as follows: Glu (OtBu), Thr (tert-butyl, tBu), and Lys (tert-butoxycarbonyl, Boc). Deprotection and cleavage of the peptides from the resin were performed with a mixture of anhydrous TFA (Wako; Osaka, Japan) (90%) and water (10%). The mixture was reacted for 1.5 h at room temperature with continuous stirring. After filtration into dry MTBE (tertiary-butyl methyl ether; Wako, Osaka Japan) under vacuum, the resin was washed with TFA. The precipitate was washed twice with MTBE and, finally, dried under vacuum.

The crude polypeptides were purified by preparative reverse-phase high-performance liquid chromatography using a Lachrom (Hitachi; Tokyo, Japan) and Develosil ODS-HG column (20 × 250 mm) 5.8 um, C18, 140 Å (Nomura Chemical Co., Ltd.; Aichi, Japan). The polypeptides were dissolved in 30% acetonitrile for the 7 a.a. peptides and 55% acetonitrile for the α3 peptide and its mutants with 0.1% TFA. They were eluted with constant percentages of each dissolved solvent. The flow rate was 10 mL/min and detection was at 220 nm. The main fractions were pooled and lyophilized. The purified polypeptides were characterized via mass spectrometry using an Axima-CFR-E (Shimadzu; Kyoto, Japan)

### 3.2. Polarized IR Microscope

For the alignments of the samples, 750 MHz nuclear magnetic resonance (Bruker, Billerica, MA, USA), equivalent to 17.4 T as the magnetic field strength, was used. The sample concentrations in the 0.1 mm inner diameter soda glass capillary (Müller, Germany) were 50 mM in ultrapure water. The sample alignments were confirmed by the polarized-light microscope Eclipse E600 POL (Nikon, Tokyo, Japan) and then each sample was lyophilized. The polarized IR measurements were performed using an FT/IR-6100 Fourier transform infrared spectrometer (JASCO, Tokyo, Japan) with an MCT detector and IRT-3000 infrared microscope accessory. The background spectra for the perpendicular alignment of the target fibril were collected in the air near the capillary and then 64 perpendicular scans from a light polarizer were measured at 4 cm^−^^1^ resolution. Next, 64 parallel scans were collected in the same condition. The background spectra for the parallel scans were measured for the last time as single-beam spectra. The resulting interferogram was Fourier-transformed using an application, Spectra manager (JASCO).

### 3.3. FT-IR Analysis

The method to evaluate the protein secondary structure from the overlapping FT-IR absorption bands was basically proposed by Aichun Dong in 1990 [33]. The bands originating from water vapor must be accurately subtracted from the protein spectrum between the 1800 and 1500 cm^−^^1^ region regardless of the baseline. Note that the IR spectra from the ATR option were adjusted in terms of intensity, for the spectra from the ATR was stronger than the infrared transmission in the low wavenumber region according to the next formula.

(1) $$S_{ATR}=k\cdot S_{T}\cdot D_{p}$$

where *S_ATR_* is a spectrum from the *ATR* option, *S_T_* is a transmission spectrum, *D_p_* is a depth of the evanescent wave, and *k* is the arbitrary coefficient. After the manipulation of the original spectra, a straight base line obtained from 2000 to 1750 cm^−^^1^ was adopted. The resultant spectra of each peptide were smoothed with a seven- or nine-point Savitzkiy–Golay function [34] to remove the white noise. The second derivative spectra are calculated by a modification of a straightforward analytical method [35]. In practice, at the data point n, the value of the second derivative *A_n_*” in absorbance units/(wavenumber)2 is

(2) $$A_{n}\prime \prime =\left(A_{n+1}-2A_{n}+A_{n-1}\right)/{\left(∆W\right)}^{2}$$

where *A_n_* is the absorbance at data point n of the original spectrum.

The strong α-helix band results in unusually strong positive lobes on each side of the α-helix band [36].

The assignment to the secondary structure of the peptides used in this work was carried out according to the datasets previously reported [15,16]. The absence of a peak around 1690 cm^−^^1^ is indicative of a parallel-type β-sheet network [37].

### 3.4. Solid-State NMR Measurement

Two methods of solid-state NMR spectroscopy, 13C CP-MAS (Cross Polarization–Magic Angle Spinning) and REDOR (Rotational Echo Double Resonance), were employed [21]. The former was used to identify the secondary structures of each 13C-labeled sample, while the latter was used to determine the distance between specific atoms. REDOR is a method for measuring the atomic distance by recoupling the weak dipole–dipole interaction between hetero-nuclei under MAS. By selectively labeling a couple of 13C and 15N atoms in a sample, it is possible to determine the distance between these atoms up to a maximum of approximately 6 Å with a resolution of about 0.1 Å.

The 13C CP-MAS and REDOR spectra were recorded at 20 °C using a Chemagnetics CMX-400 NMR spectrometer equipped with a double- and triple-resonance probe for a 5mm o.d. pencil-type spinner assembly. To avoid H1 field inhomogeneity [18], the peptide samples were placed in the central part of the spinner. The resonance frequencies for 1H, 13C, and 15N were 398.16, 100.12, and 40.35 MHz, respectively. The xy-4 pulse sequence for the irradiation of 15N was used to compensate for the error of the flip angle, off-resonance effect, and fluctuation of the H1 field [38,39]. The π pulse lengths for the 13C and 15N nuclei were 13.6 and 14.2 μs, respectively, and the proton-decoupling frequency was 70 kHz. The rotor frequency was maintained at 4000 ± 2 Hz for the REDOR experiment. The REDOR and full echo spectra were recorded at various NcTr values of 5, 10, 15, and 20 ms, where Nc and Tr were the number of the rotor cycle and rotor period, respectively. The number of transients ranged from 200 to 400 (for 5 ms), 600 to 1200 (for 10 ms), 1800 to 3600 (for 15 ms), and 5400 to10,800 (for 20 ms). The REDOR differences were evaluated as:

(3) $$S/S_{0}=\left(S_{full\;echo}-S_{REDOR}\right)/(S_{full\;echo}-S_{natural\;abundance})$$

Here, the *S_REDOR_* and *S_full echo_* are the peak intensities of the REDOR and full echo, respectively. The isotropic 13C chemical shifts were referred to the position of the Gly carboxyl group (176.03 ppm from TMS).

### 3.5. Circular Dichroism Measurements

The HDM peptide sample solution was prepared by diluting a 1 mM peptide stock solution to 10% with the buffer solution (5.56 mM HEPES and pH 7.6) to a final concentration of 100 µM. The CD measurements were performed using a J-720 circular dichroism spectrophotometer (JASCO). The spectral measurements were taken at a wavelength of 190–250 nm with a data interval of 0.2 nm, a scanning speed of 100 nm/min, and a response time of 2 s. The number of accumulations was 8 times, the bandwidth was 1 nm, and the sensitivity was 10 mdeg. A square cell with an optical path length of 1 mm was used for the measurements. The molar mean ellipticity [θ] was calculated using the following equation:

(4) $$\left[\theta \right]={\left[\theta \right]}_{obs}\cdot M_{res}/10\cdot L\cdot C$$

where [*θ*]*_obs_* is the ellipticity obtained at an observed wavelength, *M_res_* is the average molecular weight per peptide, *L* is the optical path length of the cell (mm), and *C* is the peptide concentration (g/L).

Metal ion titration using circular dichroism (CD) was performed by adding 1–5 µL aliquots of the metal ion solution (15 mM, 6 mM, 3 mM, and 1.5 mM) dropwise up to 300 µL against the 100 µM peptide solution. The resulting ellipticity at 222 nm ([θ]_222_) was plotted against the metal ion concentration, and curve fitting was performed using the following equation, assuming a 1:1 reaction between the peptide and metal ion.

(5) P+M⇄PM

(6) $$K_{B}=\left[PM\right]/[P][M]$$

where *P* and *M* represent the peptide and metal, respectively, and *K_B_* is the binding constant.

### 3.6. Atomic Force Microscopy

An atomic force microscope SPM-9500 J2 (SHIMADZU) was used for the measurements, with an NCHR-20 cantilever (Nanoworld Innovation Technology) mounted inside. The dynamic mode was employed for the measurement, which is appropriate for relatively soft samples, such as proteins and peptides. To prepare the sample, the surface of the mica substrate was cleaved off using cellophane tape. Next, 5 μL of the sample was applied to the freshly cleaved mica surface and allowed to adsorb for 1 min. The surface was then rinsed with 50 μL of pure water and dried using a gentle stream of nitrogen gas.

### 3.7. Cryo-Electron Microscopy and Image Processing

The samples for the electron microscopy of the CaRP2 and βKE peptides were prepared by adding peptide powder to ultrapure water to obtain peptide concentrations of 1 mM and 200 μM, respectively. The concentrations of the CaRP2 and βKE were determined from the absorbance measurements at 280 nm using a UV-visible absorption spectrophotometer (V-650; Jasco) and from the amount of peptide powder weighed, respectively. The sample was allowed to stand at 4 °C for 1 month for CaRP2 or at 25 °C for 1 day for βKE to allow the fibers to form. The samples used for the cryo-EM observations were prepared in the same manner. The samples for the electron microscopy of the HDM3 peptide were prepared by dissolving an appropriate amount of the peptide in ultrapure water. The pH was then adjusted to 6.0 using 0.01 M NaOH and 0.1% HCl to obtain a peptide concentration of 100 μM.

The cryo-EM observations were performed as follows: A 5 μL sample solution was applied to a glow-discharged Quantifoil holey carbon grid R1.2/1.3 Cu 200 mesh (Quantifoil Micro Tools GmbH, Groβlobichau, Germany). The grids were plunged into liquid ethane using a Vitrobot Mark IV (Thermo Fishier Scientific, Waltham, MA, USA) with a blotting time of 3.5 s at 4 °C and 100% humidity. For the CaRP2, the grid was inserted into a Titan Krios (Thermo Fishier Scientific, Waltham, MA, USA) operating at an acceleration voltage of 300 kV and equipped with a Cs corrector (CEOS, GmbH). The images were recorded with a Falcon III direct electron detector (Thermo Fisher Scientific) in the Counting mode. The data were automatically collected using EPU software (Thermo Fisher Scientific) at a physical pixel size of 1.13 Å, with 100 frames at a dose of 0.5 e-/Å per frame, an exposure time of 50 s per movie, and the defocus ranging from −0.5 to −2.0 µm. A total of 956 movies were collected.

For the βKE, the grid was inserted into a Talos Arctica (Thermo Fishier Scientific) operating at an acceleration voltage of 200 kV. The images were recorded with a K3 direct electron detector in the Counting mode. The data were automatically collected using EPU software at a physical pixel size of 1.13 Å, with 98 frames at a dose of 0.51 e-/Å per frame, an exposure time of 76.33 s per movie, and the defocus ranging from −0.6 to −2.0 µm. A total of 1056 movies were collected.

For HDM3, the grid was inserted into a Titan Krios operating at an acceleration voltage of 300 kV and equipped with a Cs corrector. The images were recorded with a Falcon III direct electron detector in the Counting mode. The data were automatically collected using EPU software at a physical pixel size of 1.13 Å, with 98 frames at a dose of 0.51 e-/Å per frame, an exposure time of 77.11 s per movie, and the defocus ranging from −0.6 to −2.0 µm. A total of 303 movies were collected.

The movie frames were subjected to beam-induced motion correction using MotionCorr2.1 [40], and the contrast transfer function (CTF) was evaluated using Gctf [41]. The motion correction and CTF estimation were processed using RELION 3.0 [42]. Using crYOLO-1.8.1 [43], the particles were segmented and extracted in square boxes of 400 pixels with 90% overlap, and those segment images were aligned and analyzed.

### 3.8. Chemical Modification of HDM1 and -2 via Diethylpyrocarbonate (DEPC)

The peptides were dissolved in a 5 mM HEPES buffer (pH 7.6) to a concentration of 200 μM. DEPC (Aldrich) was added to a final concentration of 40 mM, and the mixture was allowed to react for 1 h at 5 °C. The degree of His modification by DEPC was monitored with a UV-visible absorption spectrometer V-650 (JASCO), and the molecular weight after modification was confirmed with a MALDI-TOF mass spectrometer, AXIMA-CFR (SHIMADZU).

## 4. Conclusions

In this study, we first conducted a comprehensive structural analysis of nanofibers formed by the α3 peptide using solid-state NMR and other techniques. The results showed that the tetrameric bundle of helix molecules elongated along the fiber axis due to β-sheet-like hydrogen bonding between the N- and C-terminals of the units. Based on this structural information, we designed peptides HDM1-3 that can switch the helix fiber structure on and off depending on the presence or absence of metal ions by introducing amino acid residues with a metal-binding ability into the α3 peptide. It was found that HDM1 underwent a reversible and rapid conformational change upon the addition and removal of the metal ions. This feature is expected to be used in a variety of fields, including sensing and drug delivery systems that need to rapidly respond to environmental changes. Furthermore, peptides CaRP2 and βKE were designed to form β-sheet nanofibers. The structural and morphological observations of these peptides using cryo-EM and other techniques revealed that they formed nanofibers through the formation of β-sheet-type hydrogen bonds throughout the molecule. These examples demonstrated the relevance of applying cryo-EM techniques to engineered peptide nanofibers and provided valuable insights for future peptide design.

## Figures and Tables

**Table 1:** Names and sequences of the six peptides used in this study.

Name	N-Terminal	Sequence	C-Terminal	Number of Amino Acids
α3	H_3_N^+^	LETLAKALETLAKALETLAKA	COO^−^	21
CaRP2	H_3_N^+^	WDKDGNGTISFNE	CONH_2_	13
βKE	H_3_N^+^	KFVIFE	COO^−^	7
HDM1	H_3_N^+^	LETLAHALETLAHALETLAKA	COO^−^	21
HDM2	H_3_N^+^	LETLAKALHTLAHALETLAKA	COO^−^	21
HDM3	H_3_N^+^	LETLAKALEHLAHALETLAKA	COO^−^	21

**Table 2:** Summary of the results obtained from polarized IR spectroscopy for α3 peptide without isotope labeling.

Wave Number	Assignment	Area: Parallel	Area: Perpendicular	Dichroic Ratio	Angle (°)
1648 cm^−1^	random coil	1.66	1.12	1.48	37.3
1654 cm^−1^	α-helix	3.28	1.41	2.33	0
1660 cm^−1^	3_10_-helix or type III turn	2.21	1.99	1.11	50.2

**Table 3:** Isotope-labeled α3 peptides and their structural characteristics.

	Classification and Location	Assigned Structure */^13^C-^5^N Distance **
1L[^13^C=O], 18L[^13^C=O], or 19A[^13^C=O]	Leu or Ala, single label, and terminal region	mixture of α-helices and β-sheet
4L[^13^C=O], 8L[^13^C=O], 11L[^13^C=O], or 15L[^13^C=O]	Leu, single label, and middle region	α-helix
5A[^13^C=O], 7A[^13^C=O], 12A[^13^C=O], or 14A[^13^C=O]	Ala, single label, and middle region	α-helix
1L[^13^C=O] and A5[^15^N]	double label, N-terminal, and vicinity	4.6 (±0.1) Å
Positions of the isotope labels of α3	double label, C-terminal, and vicinity	4.5 (±0.1) Å
1L[^13^C=O] and A21[^15^N]	double label, N-terminal, and C-terminal	4.7 (±0.1) Å intermolecular
1L[^15^N] and A21[^13^C=O]	double label, N-terminal, and C-terminal	5.1 (±0.1) Å intermolecular

* The structure was assigned using the solid-state NMR: ^13^C CP-MAS. ** The distance was determined using the solid-state NMR: REDOR.

**Table 4:** Assignments of DEPC-modified sites.

Peptide	Number of DEPC	Quantity	Assignments
HDM1 (Figure 10C)	3 4	major	His, His, N-terminal His, His, N-terminal,Lys
HDM1-Ni^2+^ (Figure 10D)	0 1 2 3	major	None * Lys His, His His, His, Lys
HDM2 (Figure 10E)	3 4	major	His, His, Lys His, His, Lys, Lys

* Note that the N-terminal is blocked due to the formation of β-type intermolecular hydrogen bond.

## Data Availability

Data are contained within the article and Supplementary Materials.

## Supplementary Materials

The following supporting information can be downloaded at: https://www.mdpi.com/article/10.3390/ijms25021111/s1.
