# Supplementary material for: Structural Analyses of Designed α-Helix and β-Sheet Peptide Nanofibers Using Solid-State Nuclear Magnetic Resonance and Cryo-Electron Microscopy and Introduction of Structure-Based Metal-Responsive Properties
Source: Int J Mol Sci. 2024 Jan 16;25(2):1111. doi: 10.3390/ijms25021111 (PMC10816960; doi:10.3390/ijms25021111)
Supplement: Supplementary file 1 [file ijms-25-01111-s001.zip › ijms-2802612-supplementary.pdf]

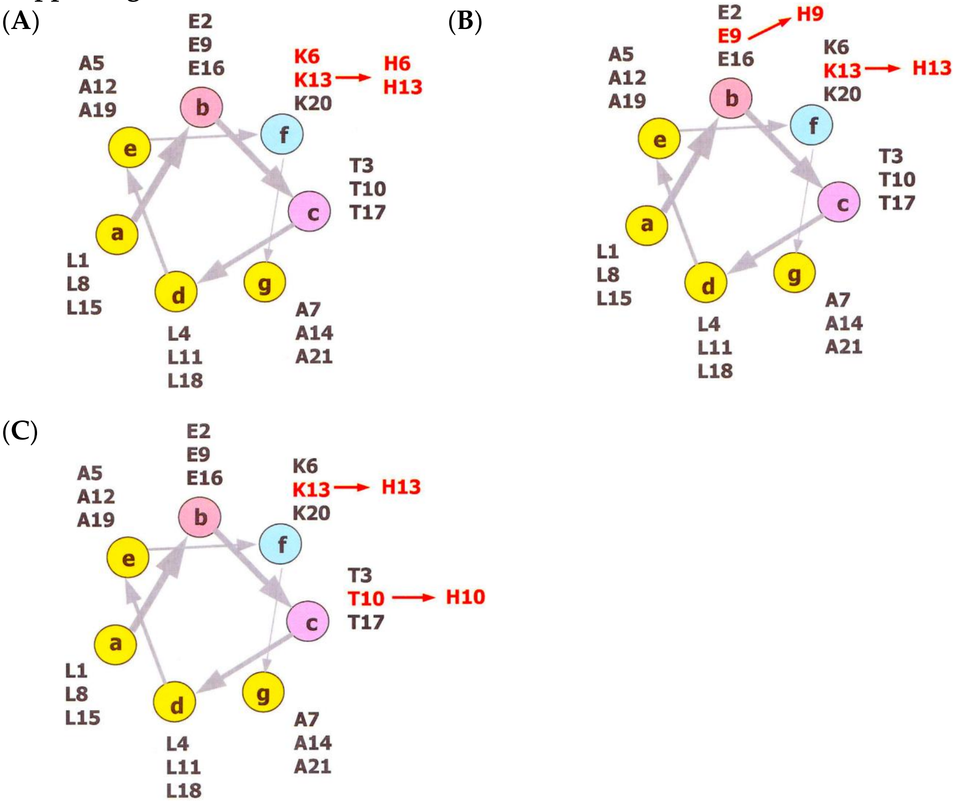

Figure S1. Helical wheel representations and amino acid substitutions of HDM1, -2, and -3. (A) HDM1, (B) HDM2, and (C) HDM3.

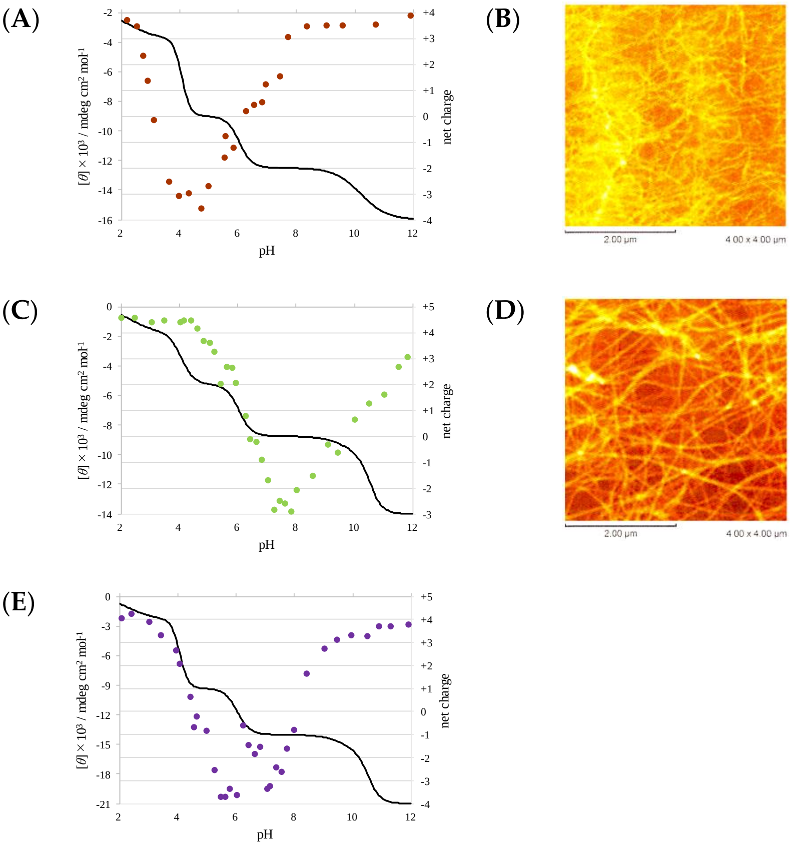

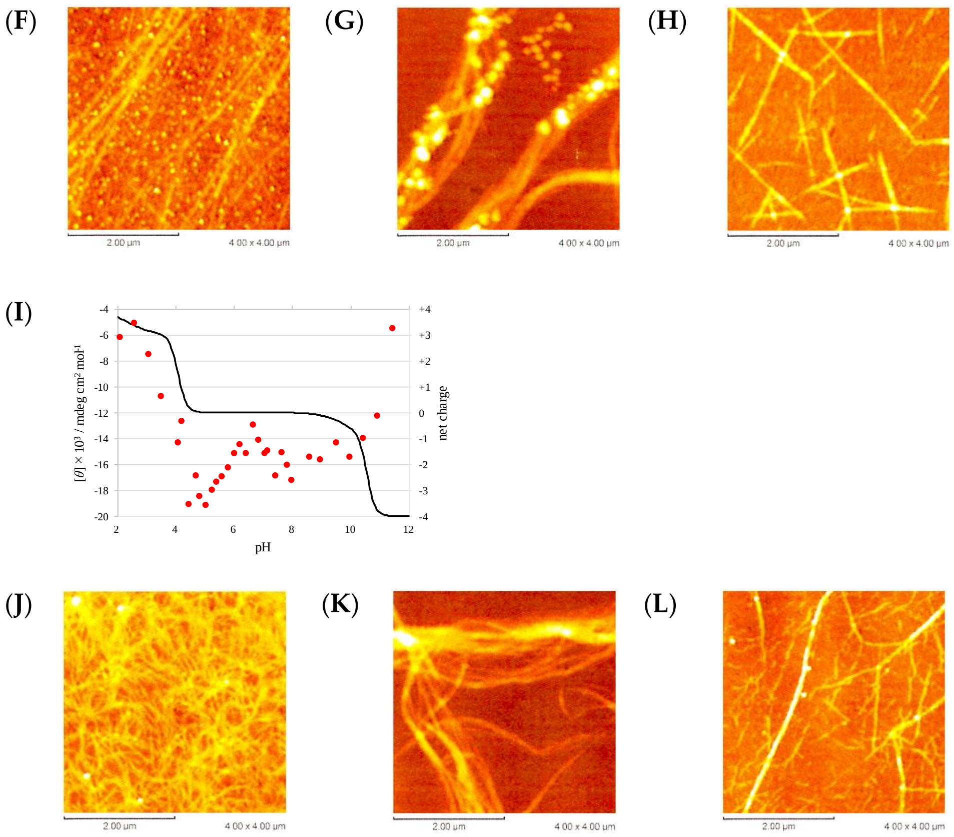

Figure S2. pH dependence of CD values and fibril morphology observed by AFM for HDM1-3 and α3. Measured θ values of CD (left axis) are shown as red dots and calculated total charge of the peptide as solid lines (right axis). It was shown that fibers are most likely to form at pH where the total charge of the peptide is close to zero, i.e., near the isoelectric point.
HDM1: (A) pH dependence of CD, and (B) AFM image at pH 4.5.
HDM2: (C) pH dependence of CD, and (D) AFM image at pH 7.6.
HDM3: (E) pH dependence of CD, and AFM images at pH 5.6 (F), pH 6.4 (G), and pH 7.6 (H).
α3: (I) pH dependence of CD, and AFM images at pH 5.0 (J), pH 6.6 (K), and pH 8.0 (L).

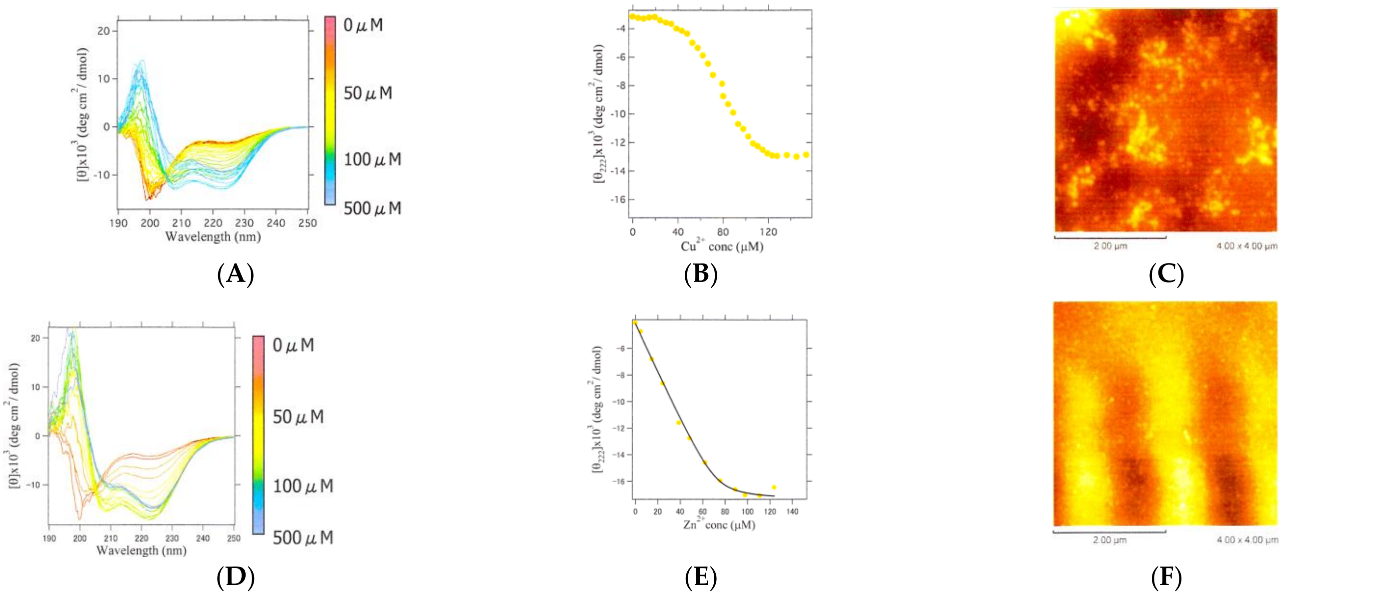

Figure S3. (A) CD spectra of HDM1 with varying concentrations of Cu^{2+}. (B) The change in the value of $[\theta]_{222}$, and (C) AFM image after addition of 100 μM of Cu^{2+} to HDM1. (D) CD spectra of HDM1 with varying concentrations of Zn^{2+}. (E) The change in $[\theta]_{222}$ and fitting by the theoretical curve. The binding constant K_B was determined to be 7.3 (±1.4) × 10^5. (F) AFM image of HDM1 after addition of 100 μM of Zn^{2+}.
